# Indications and Outcome of Carotid Endarterectomy (CEA): A Single Centre Experience

**DOI:** 10.7759/cureus.50930

**Published:** 2023-12-21

**Authors:** Mohamed S M Elshikhawoda, Sohaib Jararaa, Steven H.S. Tan, Ahmed Hashim Ahmed Mohamed, Doaa Abdalaziz Salih Abdalaziz, Abdillahi Ahmed Roble, Mahmoud Okaz, Waseem Ahmad, Abdelrhman Elsanosi, Hassan Jararah

**Affiliations:** 1 Vascular Surgery, Glan Clwyd Hospital, Rhyl, GBR; 2 Surgery, University Hospital of Wales, Cardiff, GBR; 3 General Surgery, Faculty of Medicine, The National Ribat University, Khartoum, SDN; 4 General Surgery, University Hospital of North Tees, Stock-on-Tees, GBR; 5 Vascular Surgery, University Hospital Hairmyres, Scotland, GBR

**Keywords:** stroke, transient ischemic attack, internal carotid artery stenosis, carotid endarterectomy, carotid stenosis

## Abstract

Background

Stroke is a prevalent ailment that impacts a substantial number of individuals globally, resulting in both physical impairment and mortality. One of its major causes is carotid artery stenosis. The symptoms and degree of stenosis are key indications for carotid endarterectomy (CEA). In this study, we highlight the indications and outcomes of carotid endarterectomy in our center.

Methods

This is a descriptive, retrospective, observational study. Data of patients who underwent CEA at Glan Clwyd Hospital from January 2018 to January 2023 was retrieved. The study sample consisted of patients diagnosed with symptomatic carotid artery stenosis who had CEA at Glan Clwyd Hospital. The data was analyzed using statistical software SPSS (IBM Corp. Released 2012. IBM SPSS Statistics for Windows, Version 21.0. Armonk, NY: IBM Corp).

Results

A total of 150 patients were enrolled in the study. A majority of the patients were male, accounting for 69.3% (n = 104), and had a mean age of 71.1 ± 9.9 standard deviation. A majority of the patients were smokers (48.7%) and had additional medical conditions, including hypertension (34%), ischemic heart disease (17.3%), chronic obstructive pulmonary disease (73.3%), and diabetes (46.7%). Nevertheless, the remaining comorbidities were less common. The outcome of the CEA among the patients was cardiac event 3.3% (n = 5); transient ischemic attack (TIA) 3.3% (n = 5); stroke 0.6% (n = 1); hemorrhage 2.6% (n = 4); surgical site infection 2% (n = 3); perioperative mortality 1.3% (n = 2); and cranial nerve injury 1.3% (n = 2). However, no complications were reported in most of the patients, 85.6% (n = 128).

Conclusion

An endarterectomy is quite advantageous for treating symptomatic stenosis. The findings can be applied to patients who are physically suitable for surgery. The efficacy of endarterectomy is contingent upon not only the severity of carotid stenosis but also various other parameters, such as the time elapsed between the presenting event and the surgical intervention, as well as the patient's overall medical condition. However, the CEA is the gold standard in surgical management for symptomatic carotid disease.

## Introduction

Stroke is a prevalent ailment that impacts a substantial number of individuals globally, resulting in both physical impairment and mortality [1]. The Global Burden of Disease Study has classified stroke as the second-leading cause of death globally. Around 10-15% of all stroke cases are caused by thromboembolism resulting from stenosis in the internal carotid artery (ICA) [2-3].

The North American Symptomatic Carotid Endarterectomy Trial and the European Carotid Surgery Trial, which were pivotal trials for surgery in patients with recently symptomatic ICA stenosis, demonstrated that carotid endarterectomy (CEA) led to a 7.8% decrease in the absolute risk of stroke in patients with an ICA stenosis of 50-69% and a 15.6% decrease in patients with an ICA stenosis of 70-99% when compared to conservative medical treatment [2,4,5]. Nevertheless, the advantages of surgery were contingent on the passage of time and decreased significantly with any delay [2].

Carotid endarterectomy (CEA) has been conducted since the 1950s and has been the primary treatment for individuals with symptomatic and severe asymptomatic carotid stenosis [6,7]. In this study, we are trying to highlight the indications and outcomes of carotid endarterectomy in our center.

## Materials and methods

Study design and population

This was a descriptive, retrospective, observational study. The study sample consisted of patients diagnosed with symptomatic carotid artery stenosis who had carotid endarterectomy (CEA) at Glan Clwyd Hospital. We excluded 100 patients with incomplete records or missing data.

Perioperative evaluation and management

Due to the varied referral patterns of the patients in our department, we conducted preoperative examinations using a mix of Doppler ultrasound and computed tomography angiography (CTA) to assess the degree of ICA stenosis (our institution's vascular lab does not comment on the nature or morphology of the plaque, hence the decision to go for surgery solely depends on the degree of stenosis and Rankin score, and the nature and morphology of the plaque have no impact on the decision). Following the surgery, patients were observed in the postanesthesia care unit for four hours, unless specific indications were suggesting otherwise. If the patient's hemodynamic condition remained stable, they were then transferred to the ward, and a postoperative neurological assessment was carried out. Every patient received preoperative treatment with an antiplatelet medication, either aspirin, clopidogrel, or both. Patients who were not receiving statin therapy before the operation were initiated on the medication.

Procedure

All CEAs were conducted while the patient was under general anesthesia, using magnifying loupes for enhanced visualization, and with the standard practice of inserting a shunt during the surgery. A handheld Doppler device was utilized to assess the blood flow in the ICA following shunt placement. Before cross-clamping, 5000 units of heparin were administered intravenously, and the systolic blood pressure was elevated to 150-160 mm Hg. All the arteriotomies were closed by bovine patches with a 5-0 prolene running suture. The standard policy of the department is to use the patches regardless of vessel size. Just before unclamping, systolic blood pressure was in the 100-150 mm Hg range. All CEAs were performed by the consultant or supervised by him. The total clamping time ranged from five to seven minutes.

Data collection

We retrieved the data of the patients who underwent CEA at Glan Clwyd Hospital between January 2018 and January 2023. The clinical evaluation included factors such as the patient's age, gender, comorbidities, smoking, complications, Doppler and CTA degree stenosis, length of stay (LOS), and pre-and post-operative Rankin score.

Follow up

Follow-up to assess the degree of restenosis was done after six months using the quality of life scoring system that focuses on certain factors that have different instruments (descriptions of each instrument are detailed in Appendix 1), Rankin score, and Doppler postoperatively.

In our center, all patients were followed for a duration of 10 to 12 months to assess the outcome through a face-to-face clinic and/or a telephone clinic. Assessment of the patient's experience and quality of life by assessing his postoperative complications and Rankin score (which is the standard assessment tool in most UK centers).

Data analysis

The data collected for this study was processed using SPSS software (IBM Corp. Released 2012. IBM SPSS Statistics for Windows, Version 21.0. Armonk, NY: IBM Corp) including data entry, cleaning, and analysis. Descriptive statistics were utilized to present the frequency tables with corresponding percentages. The mean and standard deviations were also reported. A bivariate analysis assessed the associations between the outcome variables and other relevant influencing factors. The statistical tests employed were the chi-square test for categorical variables and the t-test for quantitative variables. A significance level of 0.05 or less was considered statistically significant, indicating a substantial relationship between the variables.

Definitions 

Rankin Score

Documentation in the medical record of a modified Rankin score (mRS). The modified Rankin score (mRS) is a six-point disability scale with possible scores ranging from 0 to five. A separate category of six is usually added for patients who expire.

0: No symptoms.

1: No significant disability - able to carry out all usual activities, despite some symptoms.

2: Slight disability - able to look after their own affairs without assistance, but unable to carry out all previous activities.

3: Moderate disability - requires some help but can walk unassisted.

4: Moderately severe disability - unable to attend to their own bodily needs without assistance and unable to walk unassisted.

5: Severe disability - requires constant nursing care and attention, is bedridden, and is incontinent.

6: Dead.

## Results

We enrolled a total of 150 patients. Most of the patients were male, accounting for 69.3% (n = 104), and had a mean age of 71.1 ± 9.9 years standard deviation (SD).

The majority of the patients were smokers and had additional medical conditions, including hypertension (34%), ischemic heart disease (17.3%), chronic obstructive pulmonary disease (73.3%), and diabetes (46.7%). Nevertheless, the remaining comorbidities were less common (Table 1).

**Table 1:** Patient demographics and comorbidities. AF: atrial fibrillation, DM: diabetes mellitus, COPD: chronic obstructive pulmonary disease, IHD: ischemic heart disease, CCF: congestive cardiac failure, CKD: chronic kidney disease, PVD: peripheral vascular disease

		number	Percentage
Gender	Male	104	69.3%
	Female	46	30.7%
AF	Yes	5	3.3%
	No	145	96.7%
Smoking	Current smoker	73	48.7%
	Ex-smoker	53	35.3%
	Never smoked	24	16%
DM	Yes	70	46.7%
Hypertension	Yes	51	34%
COPD	Yes	110	73.3%
IHD	Yes	26	17.3%
CCF	Yes	10	3.3%
CKD	Yes	0	0
PVD	Yes	10	6.7%
Total		150	100%

The transient ischemic attack (TIA) was the most frequently recorded indication of repair, and the rest of the indications were less frequent (Table 2). Moreover, the most involved artery was the left internal carotid artery (ICA) (Table 2).

**Table 2:** Indication of CEA and site involved. CEA: carotid endarterectomy, TIA: transient ischemic attack

		Number	Percentage
Indication	TIA	75	50%
	Amaurosis Fugax	39	26%
	Stroke	58	23.3%
	Others	1	0.7%
Site			
	Right	67	44.7%
	Left	83	55.3%
	Total	222	100%

The mean time from the beginning of the symptoms to review was 3±1 days SD, and the mean time from the beginning of symptoms until operation was 9 ± 6 days standard deviation (SD). The Rankin score mean varied from 1 ± 1.1 SD preoperatively to 1.3 ± 0.57 SD postoperatively. When we compare the mean, there was no significant difference between the Rankin score in preoperative or postoperative assessment (p-value 0.229). The mean length of hospital stay postoperatively (LOS) was 2 ± 3 days SD.

Regarding the stenosis assessment, most of the patients had stenosis of 90-99% in both the Doppler US and CTA; however, there was a significant difference between the Doppler US and CTA findings (p-value 0.000) (Table 3).

**Table 3:** Co-relation between the degree of ICA stenosis in Doppler US and CTA p-value 0.000, ICA: internal carotid artery

			CTA Ipsilateral Stenosis CA		
US Ipsilateral Stenosis ICA	<50%	50-69%	70-89%	90-99%	Total
<50%	0	13	1	0	14
50-69%	0	11	5	0	16
70-89%	1	0	46	11	58
90-99%	0	2	5	53	60
Occluded	0	0	1	1	2
Total	1	26	58	65	150

The outcome of the CEA among the patients was cardiac event 3.3% (n = 5); TIA 3.3% (n = 5); stroke 0.6% (n = 1); hemorrhage 2.6% (n = 4); surgical site infection 2% (n = 3); perioperative mortality 1.3% (n = 2); and cranial nerve injury 1.3% (n = 2). However, no complications were reported in most of the patients, 85.6% (n = 128).

## Discussion

CEA remains the mainstay of treatment for carotid artery stenosis in the United Kingdom, with well-documented outcomes [8]. In this article, we report on CEA outcomes over five years with a minimum clinical follow-up of 10 months in our institutions. Most patients with symptomatic carotid stenosis received CEA surgery within the recommended timeframe, and outcomes were good, with minimal post-operative complications identified within 30 days of surgery.

Most of the patients were male, with a mean age of 71.1 + 9.9 years (SD). Nejim et al. discussed in their study that the age group that they found the most affected was between 65 and 75 years [9]. The most frequent indication, according to the study of 678 patients by Cebul et al., was TIA, followed by stroke. This finding is consistent with our study, but we don't have enough cases to determine whether there is a discrepancy [10].

According to the National Institute for Health and Care Excellence (NICE) guidelines, a stroke patient should be treated within the first 14 days from the symptom; therefore, we were committed to the guidelines with a mean of 9 + 6 days SD [11]. Regarding the stenosis assessment, most of the patients had stenosis of 90-99% in both the Doppler US and CTA, which is consistent with the literature [10,12]. However, there was a significant discrepancy between the Doppler US and CTA in assessing the degree of ICA stenosis. This might be related to the fact that the Doppler US is operator-dependent or the use of different criteria, unlike the CTA.

The outcome of the CEA among the patients was cardiac event 3.3% (n = 5); TIA 3.3% (n = 5); stroke 0.6% (n = 1); hemorrhage 2.6% (n = 4); surgical site infection 2% (n = 3); perioperative mortality 1.3% (n = 2); and cranial nerve injury 1.3% (n = 2). However, no complications were reported in most of the patients, 85.6% (n = 128).

The majority of literature indicates that problems following carotid endarterectomy (CEA) are modest. However, there have been fewer reports of complications such as cardiac events, postoperative transient ischemic attacks (TIAs), strokes, hemorrhages, and surgical site infections [4,5,7].

Lamba et al. concluded in their study that he got 1% postoperative stroke and 2.6 with TIA [12]. This discrepancy might be due to the long-term follow-up, the number of patients, and the study type. Yei et al. shed light on the patient's mortality and stroke rate when comparing the CEA versus the stenting. They stated that CEA is associated with lower mortality, longer survival, and fewer strokes in comparison to carotid stenting, which aligned with our study [13]. Cooper et al. had contradictory results of higher mortality, stroke rates, and cardiac events. This is because their study focused on patients on hemodialysis [14].

Our study consisted of patients with cranial nerve injury, mainly hypoglossal neuropraxia, where patients recovered within two to three weeks. Moreover, we had a perioperative mortality rate of 1.3%. This is because the patients had adverse cardiac events such as myocardial infarction and did not survive.

We evaluated the preoperative and postoperative Rankin scores of the patients to assess their neurocognitive function postoperatively. The result was that there was no significant difference in the preoperative and postoperative Rankin scores. However, other authors agreed that there was an improvement. This difference is because they used more accurate assessment tools other than the clinical Rankin score [15-17].

Limitations

The limitations of this study include its reliance on data from a single center and the retrospective nature of data collection. Hence, our analysis was limited to the data in the patient's records, without any subsequent monitoring over an extended period. Furthermore, we eliminated a substantial proportion of patients from the study due to their insufficient medical records, and we lacked data for individuals who did not exhibit symptoms at all.

## Conclusions

An endarterectomy is quite advantageous for treating symptomatic stenosis. The findings can be applied to patients who are medically suitable for surgery. The efficacy of endarterectomy is contingent upon not only the severity of carotid stenosis but also various other parameters, such as the time elapsed between the presenting event and the surgical intervention, as well as the patient's overall physical condition. However, the CEA is the gold standard in surgical management for symptomatic carotid disease.

## Appendices

Appendix 1 

**Table 4:** Description of Quality of Life Scoring systems SF-36: medical outcomes short form 36; SIP: sickness impact profile; HAD: hospital anxiety and depression scale; ADL: Katz index of independence in activities of daily living; EQ-5D: European quality of life EQ-5D questionnaire; MILQ: multidimensional index of life quality questionnaire, and the Likert scale.

System	Components	
Likert Scale^1,2^	5 or 7-point scale used to measure how much an individual agrees or disagrees with statements about quality of life.	
	Agreement, frequency, importance, and likelihood are taken into account.	
SF-36^3^	36 items measuring eight conceptual domains or dimensions of health: (higher score = better outcome).	
	General health (GH)	Measurement of perceived overall health, including past and present health.
	Physical functioning (PF)	Indicates the level of limitations in lifting, bending, kneeling, or walking a moderate distance.
	Bodily pain (BP)	Represents the intensity, frequency, and duration of bodily pain and limitations in normal activities due to pain.
	Mental health (MH)	Measures the emotional, cognitive, and intellectual status of the patient.
	Role physical (RP)	Measures the degree in performing of usual activities for age and social status.
	Role emotional (RE)	Measures personal feelings of job performance at work or other activities.
	Vitality (VT)	Measures feelings of energy, fatigue, and tiredness.
	Social functioning (SF)	Indicates ability to develop and maintain mature social relationships.
	The SF-36 scores can be related to activities of daily living. For example, 80% of responders who judged their general health as being good, very good, or excellent had a score of 61 on the general health scale of SF-36.^10^	
	Note: Both SF-36 and SF-12 surveys can provide two summary measures – Physical Component Score (PCS) and Mental Component Score (MCS)	
SIP^4,5^	12 items assessing physical and psychological domains. Higher scores indicate worse outcomes.	
	Physical	Ambulation, mobility, body care, movement
	Psychosocial	Social interaction, communication, alertness behavior, emotional behavior, sleep, eating, home management, reaction, pastimes, employment.
HAD^6^	14 items scale to determine levels of anxiety and depression	
	Anxiety	Feeling tense, frightened, worried, relaxed, restless, panicked.
	Depression	Enjoyment, laugh, cheerfulness, slowed down, interest in appearance, enjoy hobbies.
ADL^7^	Assessment of dependency and functional skills	
	Bathing, dressing, toileting, transfers, continence, feeding, cleaning, food shopping, public transportation, cooking.	
MILQ^8^	35 items covering satisfaction with nine domains.	
	Satisfaction with mental health, physical functioning, physical health, cognitive functioning, intimacy, social functioning, productivity, relationship with health professionals, and financial status.	
EuroQOL^9^ (EQ-5D)	5 domains of EQ-5D index:	
	Mobility	Ability to walk.
	Self-care	Ability to dress.
	Usual activities	Activities of daily living.
	Pain/Discomfort	Level of pain.
	Anxiety/Depression	Level of anxiety or depression.

References

1. Cohen DJ, Stolker JM, Wang K, Magnuson EA, Clark WM, Demaerschalk BM, Sam AD, Jr., Elmore JR, Weaver FA, Aronow HD, Goldstein LB, Roubin GS, Howard G, Brott TG. Health-related quality of life after carotid stenting versus carotid endarterectomy: Results from crest (carotid revascularization endarterectomy versus stenting trial). Journal of the American College of Cardiology. 2011;58:1557-1565

2. Stolker JM, Mahoney EM, Safley DM, Pomposelli FB, Jr., Yadav JS, Cohen DJ. Health-related quality of life following carotid stenting versus endarterectomy: Results from the sapphire (stenting and angioplasty with protection in patients at high risk for endarterectomy) trial. JACC. Cardiovascular interventions. 2010;3:515-523

3. Ware JE, Jr., Sherbourne CD. The mos 36-item short-form health survey (sf-36). I. Conceptual framework and item selection. Medical care. 1992;30:473-483

4. Bergner M, Bobbitt RA, Carter WB, Gilson BS. The sickness impact profile: Development and final revision of a health status measure. Medical care. 1981;19:787-805

5. de Bruin AF, de Witte LP, Stevens F, Diederiks JP. Sickness impact profile: The state of the art of a generic functional status measure. Soc Sci Med. 1992;35:1003-1014

6. Lewis G, Wessely S. Comparison of the general health questionnaire and the hospital anxiety and depression scale. The British journal of psychiatry : the journal of mental science. 1990;157:860-864

7. Katz S, Downs TD, Cash HR, Grotz RC. Progress in development of the index of adl. The Gerontologist. 1970;10:20-30

8. Avis NE, Smith KW, Hambleton RK, Feldman HA, Selwyn A, Jacobs A. Development of the multidimensional index of life quality. A quality of life measure for cardiovascular disease. Medical care. 1996;34:1102-1120

9. Euroqol--a new facility for the measurement of health-related quality of life. Health Policy. 1990;16:199-208
